# Condensation and Protection of DNA by the *Myxococcus xanthus* Encapsulin: A Novel Function

**DOI:** 10.3390/ijms23147829

**Published:** 2022-07-15

**Authors:** Ana V. Almeida, Ana J. Carvalho, Tomás Calmeiro, Nykola C. Jones, Søren V. Hoffmann, Elvira Fortunato, Alice S. Pereira, Pedro Tavares

**Affiliations:** 1 UCIBIO-Applied Molecular Biosciences Unit, Department of Chemistry, NOVA School of Science and Technology, Universidade NOVA de Lisboa, 2829-516 Caparica, Portugal; acv.almeida@campus.fct.unl.pt (A.V.A.); ad.carvalho@campus.fct.unl.pt (A.J.C.); 2 Associate Laboratory i4HB-Institute for Health and Bioeconomy, NOVA School of Science and Technology, Universidade NOVA de Lisboa, 2829-516 Caparica, Portugal; 3 CENIMAT/i3N, Department of Materials Science, NOVA School of Science and Technology, Universidade NOVA de Lisboa, 2829-516 Caparica, Portugal; t.calmeiro@campus.fct.unl.pt (T.C.); emf@fct.unl.pt (E.F.); 4 ISA, Department of Physics and Astronomy, Aarhus University, 8000 Aarhus C, Denmark; nykj@phys.au.dk (N.C.J.); vronning@phys.au.dk (S.V.H.)

**Keywords:** encapsulin, protein nanocages, DNA binding and protection, DNA condensation, EMSA, thermal stability, SRCD, AFM

## Abstract

Encapsulins are protein nanocages capable of harboring smaller proteins (cargo proteins) within their cavity. The function of the encapsulin systems is related to the encapsulated cargo proteins. The *Myxococcus xanthus* encapsulin (EncA) naturally encapsulates ferritin-like proteins EncB and EncC as cargo, resulting in a large iron storage nanocompartment, able to accommodate up to 30,000 iron atoms per shell. In the present manuscript we describe the binding and protection of circular double stranded DNA (pUC19) by EncA using electrophoretic mobility shift assays (EMSA), atomic force microscopy (AFM), and DNase protection assays. EncA binds pUC19 with an apparent dissociation constant of 0.3 ± 0.1 µM and a Hill coefficient of 1.4 ± 0.1, while EncC alone showed no interaction with DNA. Accordingly, the EncAC complex displayed a similar DNA binding capacity as the EncA protein. The data suggest that initially, EncA converts the plasmid DNA from a supercoiled to a more relaxed form with a beads-on-a-string morphology. At higher concentrations, EncA self-aggregates, condensing the DNA. This process physically protects DNA from enzymatic digestion by DNase I. The secondary structure and thermal stability of EncA and the EncA−pUC19 complex were evaluated using synchrotron radiation circular dichroism (SRCD) spectroscopy. The overall secondary structure of EncA is maintained upon interaction with pUC19 while the melting temperature of the protein (*T*_m_) slightly increased from 76 ± 1 °C to 79 ± 1 °C. Our work reports, for the first time, the in vitro capacity of an encapsulin shell to interact and protect plasmid DNA similarly to other protein nanocages that may be relevant in vivo.

## 1. Introduction

Spatial organization is an essential mechanism used by microorganisms to balance the complexity of intracellular biochemical reactions. Due to the absence of intracellular lipid-based membranes in prokaryotes, protein nanocages emerged as a solution to maintain distinct controlled environments [1].

Encapsulins are hollow icosahedral protein nanocages found in Bacteria and Archaea, with a wide range of sizes resulting from the self-assembly of identical monomers into either 24, 32, or 42 nm wide structures, composed of 60, 180, and 240 subunits, respectively [2].

The function of encapsulins is related to the type of cargo protein encapsulated in the protein shell. The mechanism of encapsulation of the cargo proteins occurs through the spontaneous self-assembly of encapsulin cargo complexes by the co-expression of the encapsulin shell monomers and both native or artificial cargo proteins presenting a C-terminal targeting peptide [3].

A total of four encapsulin subfamilies are described. The cargo proteins from the classical or family 1 are related to known redox proteins: dye-decolorizing peroxidase (Dyp), ferritin-like proteins (Flp) and iron-mineralizing encapsulin-associated Firmicutes (IMEF), ferredoxin, and nitrite reductase hydroxylamine (NIR-HAO) [4,5,6,7,8,9].

*Myxococcus (M.) xanthus* is used as a model organism for cooperative social multicellular development usually associated with more complex eukaryotic cells. Upon nutrient starvation, the *M. xanthus* rod-shaped cells interact and aggregate, forming fruiting body structures. For this process, gene expression and cell movements are strictly regulated [10]. The *M. xanthus* organism produces an encapsulin system composed of an encapsulin shell protein (EncA), 32 nm wide (180-mer), and three cargo proteins: EncB and EncC, both containing ferritin-like domains with a highly conserved EXXH iron binding motif (X represents any amino acid residue), and a third cargo protein, EncD, with an unknown function. As such, the *M. xanthus* encapsulin is suggested to function as a major iron storage container with a capacity to accommodate ~30,000 irons [11].

The encapsulins have a relatively high structural homology and most likely share a common ancestor with the viral capsid of HK97-type viruses, which encapsulate double stranded DNA [12]. In fact, results from mutational studies of the viral capsid proteins resulted in proteins with smaller dimensions and an inability to harbor DNA, similar to the encapsulin proteins. The accumulation of five mutations lead to the formation of smaller phage capsids, resembling the size and geometry of encapsulins with a diameter of 35 nm, while other mutations blocked native viral capsid expansion during the maturation process, maintaining the same diameter and structure, analogous to the encapsulin shell [13]. Moreover, substitution of specific key residues involved in the maturation process of the capsid caused the loss of the ability to pack and retain DNA [14]. Similar evolutionary divergence might explain the differences between Caudovirales and encapsulins.

Besides Caudovirales proteins, other protein cages are described to interact with DNA. DNA-binding protein from starved cells (Dps) bind to linear or supercoiled plasmid and genomic DNA through the protein surface. Although the external surface of most Dps proteins has a global negative charge, electrostatic interactions between the DNA and specific positive residues (lysine or arginine) in the N- and C-terminal tails of the protein allow the binding, condensation, and protection of DNA against chemical and physical damage [15,16,17]. No specificity between Dps and DNA sequences are reported so far. Contrary to what was believed, a recent work revealed that, in the Dps−DNA complex, DNA is not wrapped around the Dps molecule, contrasting with the histone-like DNA binding mode [18]. Partial deletion of lysine residues at the N-terminal extensions impairs DNA condensation, while removal of all these residues impairs DNA interaction [19]. Additionally, changes in the overall charge of the tail by replacing a neutral residue with a negative one (lowering the pI of the tail from 8.2 to 5.9) impaired the DNA interaction in the *Marinobacter hydrocarbonoclasticus* Dps [20].

In this work, we investigated the binding of the encapsulin shell (EncA) from *M. xanthus* (a member of the classical encapsulin subfamily) to supercoiled plasmid DNA (pUC19), both in the absence and presence of its native cargo protein EncC, a ferritin-like protein, using electrophoretic mobility shift assays (EMSA), and atomic force microscopy (AFM). The DNA protective effect was also studied using the DNase protection assay. Furthermore, we assessed the changes in the secondary structure and thermostability of the EncA bound to pUC19. Our results suggest a novel in vitro function of the encapsulin shell besides encapsulation of native or artificial cargo proteins: the ability to interact and protect plasmid DNA, probably by a mechanism similar to other known protein nanocages.

## 2. Results

### 2.1. Expression and Characterization of the M. xanthus Encapsulin System

The encapsulin shell protein (EncA), its ferritin-like cargo protein (EncC), and the encapsulin shell harboring the Flp-type cargo protein (EncAC) from *M. xanthus*, were produced by heterologous expression in *Escherichia (E.) coli*. The recombinant proteins were produced as soluble proteins and purified to homogeneity using a combination of chromatographic steps.

**Figure 1:**
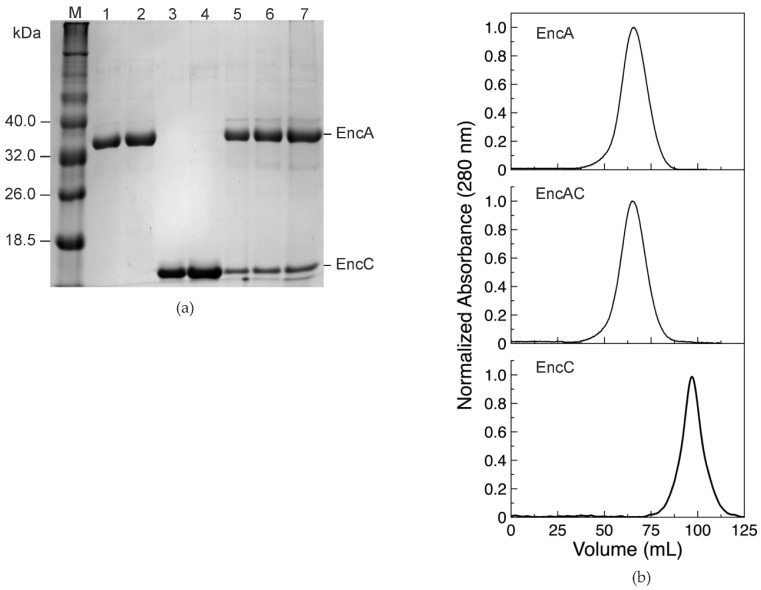
Overexpression and purification of encapsulins and the EncC cargo protein. (**a**) SDS-PAGE gel of pure EncA protein shell, EncC cargo protein, and co-expressed EncAC system. M—LMW II protein marker (NZYTech), 1 and 2—1.5 µg and 3.5 µg of EncA; 3 and 4—1.5 µg and 3.5 µg of EncC; 5 to 7—serial dilution of EncAC complex. EncA and EncC bands are labelled. (**b**) size exclusion chromatography analysis using a Sephacryl S-500 column equilibrated in 200 mM MOPS buffer, pH 7.0, 200 mM NaCl of EncA, and a EncAC complex and EncC protein.

The EncA shell and the EncC cargo protein present a single pure band in the SDS-PAGE gel, consistent with the expected apparent molecular mass of the monomers (32.5 and 12.8 kDa, for the deduced amino acid sequences) (Figure 1a). The EncAC complex displays two electrophoretic bands under denaturation conditions, corresponding to the EncA and EncC proteins (Figure 1a). Size exclusion chromatography of the co-expressed EncAC complex, in native conditions, reveals a single peak with an elution volume identical to the EncA (Figure 1b), suggesting the encapsulation of the EncC cargo protein into the EncA nanocage. Additionally, the pure EncA and EncAC proteins present hydrodynamic diameters of 35 ± 1 nm and 40 ± 3 nm, as measured by dynamic light scattering (DLS), in agreement with the atomic structure of the 180-mer EncA shell and EncA harboring EncC protein. These data indicate that both proteins self-assembled correctly and support the encapsulation of the EncC cargo protein within the EncA protein [11,21].

Recombinant protein quantification by densitometry analysis of SDS-PAGE gels (Figure 1a) accounts for 55 ± 4% of EncA monomers in the complex, which corresponds to an assembly of 180 monomers of EncA carrying an average of 150 ± 7 EncC monomers per cage.

### 2.2. Characterization of the DNA Binding Properties of Encapsulins

**Figure 2:**
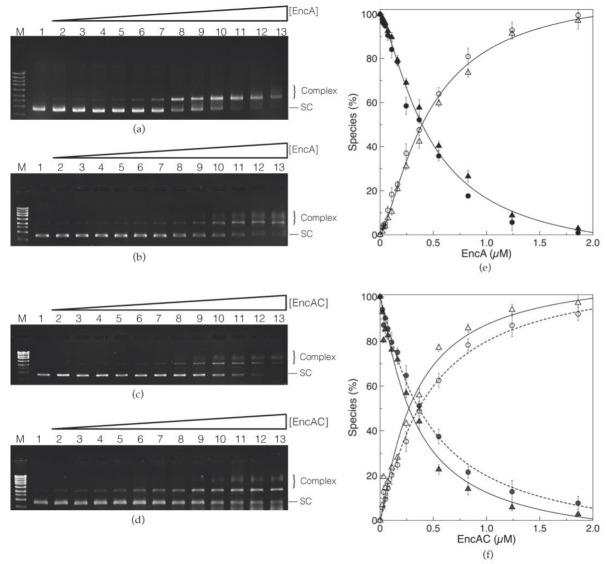
Binding of encapsulin to supercoiled pUC19 (5 nM) in high and low ionic strength conditions. (**a**) EMSA of EncA in 50 mM MOPS buffer, pH 7.0, 50 mM NaCl, and (**b**) 200 mM MOPS buffer pH 7.0, 200 mM NaCl. (**c**) EMSA of EncAC complex in 50 mM MOPS buffer, pH 7.0, 50 mM NaCl, and (**d**) 200 mM MOPS buffer pH 7.0, 200 mM NaCl. M—NZYLadder II; 1 to 13—binding of EncA and EncAC to the plasmid DNA with increasing protein concentrations: 0, 0.02, 0.03, 0.05, 0.07, 0.11, 0.16, 0.24, 0.37, 0.55, 0.83, 1.24, and 1.86 µM. The free form of the supercoiled plasmid pUC19 (SC) and the protein−pUC19 complexes bands (complex) are labelled. Hill plots of DNA binding to (**e**) EncA and (**f**) EncAC from densitometric analysis of three sets of experiments in either 50 mM MOPS buffer pH 7.0, 50 mM NaCl (circles) or 200 mM MOPS buffer, pH 7.0, 200 mM NaCl (triangles). Free DNA is plotted as full and the complex as empty circles or triangles. The lines on top of the experimental data are theoretical fits using Equation (1). For the binding of (**e**) EncA to pUC19, a single set of parameters, *K*_D_ and *n*, was used for the theoretical fit, whereas for the (**f**) EncAC complex, two sets of parameters were employed for the binding reaction in low (dashed lines) and high (full lines) ionic strength conditions.

The ability of encapsulins to bind circular double stranded plasmid DNA was evaluated using electrophoretic mobility shift assays (EMSAs). The EMSAs were performed using the supercoiled form of the pUC19 plasmid (the electrophoretic pattern of the pUC19 in different conformations is presented in Figure S1, Supplementary Materials) in low (50 mM MOPS buffer, pH 7.0 with 50 mM NaCl) and high (200 mM MOPS buffer, pH 7.0, 200 mM NaCl) ionic strength conditions. The results indicate that EncA is capable of binding to the supercoiled form of pUC19. Binding of EncA to the plasmid DNA leads to the formation of EncA−pUC19 complexes that migrate slower when compared with the free supercoiled form (Figure 2a). Increasing the protein concentration upshifts the supercoiled pUC19 band to the relaxed form of the plasmid, with an apparent saturation at around 2 µM of EncA. Furthermore, at protein concentrations higher than 0.83 µM, a smear with smaller electrophoretic mobility is noticed, as well as a loss in the total intensity of the bands (between 15% and 20% of the initial intensity of the supercoiled pUC19 band is no longer detected). This observation can be rationalized as follows: (i) At higher protein concentrations, the encapsulin condensates the DNA forming large complexes that do not enter in the gel and are lost during gel manipulation; and (ii) the GreenSafe dye used for detection is not able to interact with the large, compact, protein–DNA complexes. Indeed, most of these commercial dyes bind to DNA by intercalation and/or electrostatic interactions with the negative phosphate groups in DNA [22,23], and thus, in the encapsulin–DNA complexes, these are in part unavailable (due to interactions with the protein), weakening the detection. Additionally, the formation of EncA−pUC19 complexes was not affected by increasing the ionic strength in the binding reaction (Figure 2b). At both ionic strengths, the formation of the complex was plotted and fitted with the Hill equation (Equation (1), Section 4.4) (Figure 2e). Under these conditions, the apparent dissociation constant (*K*_D_) was 0.3 ± 0.1 µM for the low and high ionic strength conditions with a Hill coefficient (*n*) of 1.4 ± 0.1, indicating a moderate positive cooperativity on DNA binding, similar to the ones reported for proteins from the ferritin family, such as *Desulfovibrio vulgaris* Dps-like bacterioferritin (*n =* 1.3 ± 0.2), Dps proteins from *Marinobacter hydrocarbonoclasticus* (*n* = 1.2 ± 0.1), or *Deinococcus radiodurans* (*n* = 1.3 ± 0.2) binding to supercoiled plasmid DNA [20,24,25].

Contrary to EncA, the cargo protein alone (EncC) was not able to bind to supercoiled pUC19 (Figure S2). Yet, the DNA binding capacity of the EncAC complex was comparable to EncA, leading to the formation of EncAC−pUC19 complexes with similar electrophoretic migration profiles (Figure 2c,d). As for the EncA protein, the interaction between the EncAC complex and supercoiled pUC19 was not affected by the increase in the ionic strength (Figure 2d), resulting in a *K*_D_ of 0.4 ± 0.1 µM and 0.3 ± 0.1 µM in low and high ionic strength buffers, and an *n* value of 1.1 ± 0.1 for both buffer conditions (Figure 2f). As such, the interaction with DNA seems specific to EncA and not affected by the presence of the EncC cargo protein inside its cavity.

The pUC19 band shift observed in the EMSA assays from the supercoiled conformation to a form that migrates slower in the gel when incubated with the encapsulin proteins can either be explained by the relaxation of the plasmid DNA topology and/or to a mechanism of DNA condensation associated with protein self-aggregation. Both processes were previously described for Dps protein nanocages binding to DNA [17].

**Figure 3:**
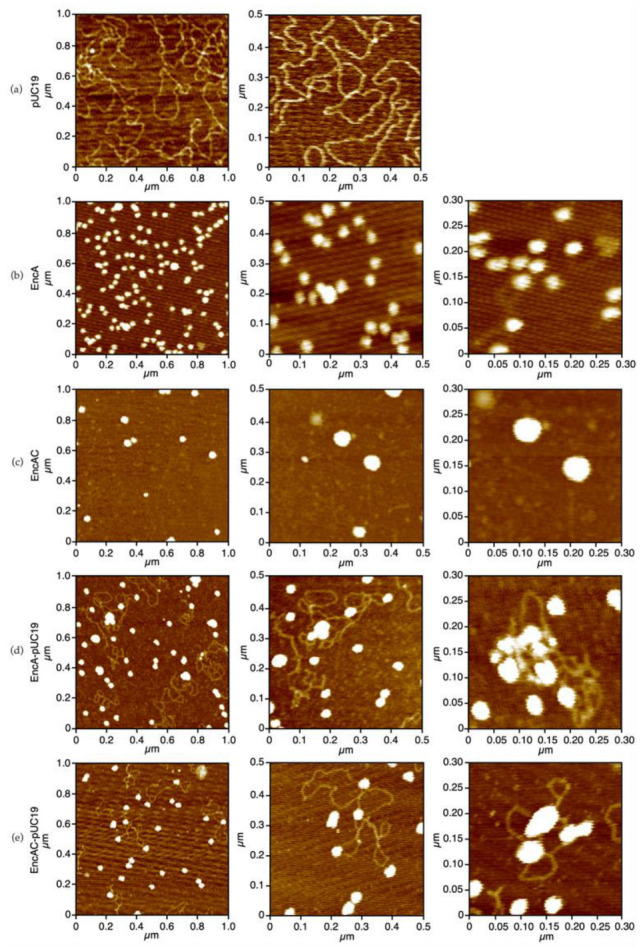
AFM imaging of the encapsulins binding to supercoiled DNA. (**a**) pUC19; (**b**) EncA; (**c**) EncAC; (**d**) EncA−pUC19 complexes; and (**e**) EncAC−pUC19 complexes. All samples were buffered in 50 mM MOPS, pH 7.0 containing 50 mM NaCl and 5 mM NiCl_2_.

The topology of the protein–DNA complexes was characterized by atomic force microscopy (AFM) and compared with the free forms of the proteins and plasmid DNA (Figure 3). The micrographs in Figure 3a show that pUC19 is predominantly in the supercoiled form, with strands crossing (bright spots on the DNA molecule). The EncA protein molecules display a uniform spherical shape (Figure 3b), approximately 35 nm wide, in agreement with the diameter determined for the atomic structure (PDB: 4PT2 and 7S20) [11,21]. The images of the EncAC complex show larger particles, probably owing to a broadening effect due to AFM tip-sample convolution effects (Figure 3c). Binding of both EncA and EncAC proteins to the plasmid DNA caused the formation of larger protein−DNA condensates (Figure 3d,e). In the presence of DNA most protein molecules appear as very large aggregates surrounded by DNA loops. In some images, mostly for the EncAC−pUC19 complexes, the spherical protein particles are randomly bound to the DNA molecules (Figure 3e, left image) with a “beads-on-a string” morphology.

### 2.3. DNA Protection Assays

**Figure 4:**
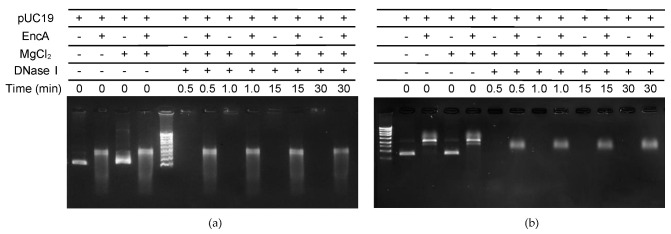
Protective effect of the EncA−pUC19 complex against DNase I digestion. Controlled digestion (**a**) in 50 mM MOPS, pH 7.0, 50 mM NaCl and (**b**) in 200 mM MOPS, pH 7.0, 200 mM NaCl. pUC19 (5 nM), either free or pre-incubated with EncA (1.24 µM), in the presence of 5 mM MgCl_2_ was reacted with 1.2 µg/µL of DNase I at room temperature. The digestion was monitored after 0.5, 1.0, 15, and 30 min of hydrolysis reaction. The composition of each reaction is described above the gel.

To study the protective effect of encapsulins, controlled digestion of pUC19 with DNase I was tested in different conditions. The incubation of free pUC19 with DNase I leads to its complete hydrolysis within less than 1 min, regardless of the ionic strength of the buffer used in the reaction (Figure 4). When pUC19 was pre-incubated with 1.24 µM of EncA, and subsequently digested with DNase I, approximately 15% of the supercoiled DNA was hydrolyzed after 1 min in low ionic strength (Figure 4a) and 35–40% at high ionic strength (Figure 4b). For longer reaction times (30 min) the intensity of the pUC19 band decreased by around 55% at low ionic strength, indicating a considerable degree of digestion, whereas no further hydrolysis was observed at high ionic strength. These results demonstrate that the binding of encapsulin to the pUC19 molecules physically shields the DNA from DNase enzymatic digestion. The different results obtained for both buffer conditions may be explained the level of looping and compaction of the protein–DNA condensates. The present data suggests that in the experimental conditions tested, higher concentrations of salts favor more compact aggregates, which might be physiologically relevant as part of a response mechanism in salt stressful conditions.

### 2.4. Secondary Structure Assessment and Thermal Stability

**Figure 5:**
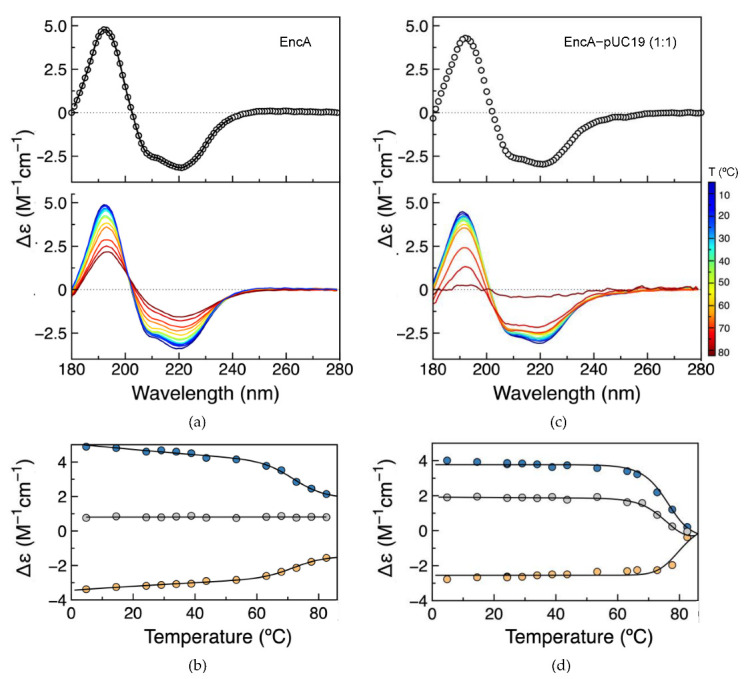
Secondary structure and thermostability assessment of EncA and EncA−pUC19 complex by SRCD. (**a**) Spectra of EncA (top, open circles) with spectral reconstructed data from Dichroweb analysis (solid line) and temperature scans of EncA (bottom). (**b**) EncA thermal denaturation monitorization. (**c**) Spectra of EncA–pUC19 (top, open circles) and temperature scans (bottom). (**d**) EncA–pUC19 thermal denaturation monitorization. The EncA and EncA–pUC19 thermal denaturation was monitored at 192 nm (blue), 220 nm (yellow), and 202 nm (grey), and the solid lines overlaying the experimental data result from a non-linear least-squares fit to the data based on a two-state denaturation model (Equation (2)).

The AFM data presented above demonstrate the changes in the morphology of pUC19 in the presence of encapsulin proteins (free or with its native cargo protein) and the aggregation of protein molecules in the protein–DNA complexes. In parallel, the secondary structure and thermal stability of encapsulins, free and bound to plasmid DNA, were assessed by synchrotron radiation circular dichroism (SRCD). The spectra of the free EncA and EncA−pUC19 complex are nearly identical (Figure 5a,c), with negative peaks at 209 and 220, and a positive peak at 192 nm, consistent with a folded protein. The small spectral differences between the EncA and the EncA–pUC19 are justified by the spectral contribution of pUC19. A more detailed analysis of the secondary structure of EncA was accomplished by spectral deconvolution using DichroWeb online tools [26] and compared with the secondary structure content of its atomic structure (PDB: 4PT2 and 7S20) using 2Struc, with agreeable results (Table 1) [27].

Temperature scans from ~5 to 83 °C were performed to compare protein folding and thermal stability of the EncA and EncA−pUC19 complex (Figure 5a,c). In both cases, increasing the temperature does not affect the spectral shape, but decreases the CD signal intensity, more extensive in the EncA−pUC19 sample, evidencing the loss of secondary structure. Following the denaturation process at three distinct wavelengths (192, 220, and 202 nm) and fitting the experimental data with a two-state thermal denaturation equation (Equation (2), Section 4.6) shows that EncA presents high thermal stability with a melting temperature (*T*_m_) of 76 ± 1 °C (Figure 5b), but nevertheless lower than the *T*_m_ value of 86.6 °C reported for the *Quasibacillus thermotolerans* encapsulin [28]. In the presence of pUC19, the *T*_m_ increases to 79 ± 1 °C, showing a slight increase in the thermostability of the protein when complexed to DNA (Figure 5d). Furthermore, a 4-fold increase in the enthalpy change at the unfolding transition (∆*H*_m_) was determined for the EncA−pUC19 complex when compared with the free encapsulin (184 ± 5 vs. 45 ± 2 kcal/mol). This difference may be explained by a higher number of non-covalent molecular interactions between EncA and the pUC19 molecule, as well as between aggregated protein molecules in the condensates, requiring more energy to disrupt the complexes. Furthermore, the final denatured form of the EncA−pUC19 complex may have a less favorable hydration than the EncA one. The latter hypothesis is supported by the different final denaturated state spectra obtained for EncA and EncA−pUC19. Contrary to what is observed for the denatured form of EncA, there is a drastic loss of ellipticity from 180 to 280 nm, pointing to the complete loss of secondary structure elements for the EncA−pUC19 complex. It is thus probable that the denaturated state will be less prone to hydration.

## 3. Discussion

To our knowledge, we are reporting, for the first time, the capacity of an encapsulin cage protein to bind and protect circular double stranded DNA. The electrophoretic mobility shift assays (EMSA) revealed that the EncA protein was able to bind the supercoiled plasmid DNA pUC19 with an apparent dissociation constant of 0.3 ± 0.1 µM and a Hill coefficient of 1.4 ± 0.1, similarly to other protein nanocages [20,24,25]. The native cargo protein of this encapsulin system, EncC, did not bind to pUC19 under the conditions tested. However, when encapsulated within the EncA shell, the EncAC complex displayed a DNA−binding capacity similar to the EncA protein (*K*_D_ = 0.4 ± 0.1 µM and 0.3 ± 0.1 µM for low and high ionic strengths, respectively, and *n* = 1.1 ± 0.1), suggesting that the overall electrostatic charge and binding properties of the outer surface was not affected by the encapsulation process. Increasing the NaCl concentration of the binding buffer did not significantly affect the electrostatic interaction between the protein and DNA.

The data presented here seems to indicate that in a first stage, binding of EncA converts the plasmid DNA into a more relaxed form, with the protein molecules appearing along the pUC19 molecules as “beads-on-a-string”, as revealed by the AFM images. At higher protein concentrations the protein molecules bound to the DNA self-aggregate, looping and compacting the DNA. This mechanism was described for the *E. coli* Dps, a nucleoid-associated protein, that binds DNA non-specifically, promoting its condensation through a protein self-aggregation process [17]. Furthermore, the binding of encapsulin protects the DNA from enzymatic digestion by DNase I, which suggests a protective effect caused by physical shielding.

The interaction between DNA and the outer surface of protein cages is relatively well characterized in Dps proteins and, in most cases, is mediated by positively charged amino acid residues, lysine, and arginine present in the protruding N- or C- terminal flexible extensions [15,20,29,30]. However, in encapsulin proteins, the N-terminal regions of each monomer are positioned towards the cavity, interacting with the cargo protein, while the C-termini are part of the pore architecture and thus inaccessible for interaction. The encapsulin outer surface, for instance, in the Dps proteins, is predominantly negatively charged, yet two distinct positively charged clusters are present. The first one, located within the monomer, is composed of three lysines and three arginines, and the second comprises four lysine and four arginine residues at each dimer interface (see Figure S3). Although these regions are located at the external surface of the encapsulin shell, they are less solvent accessible than the N- and C- terminal tails of Dps proteins. Notwithstanding, in the case of the *Helicobacter pylori* Dps, DNA interaction occurs through the positive protein surface [31].

Additionally, the overall secondary structure content of EncA is not greatly affected by DNA binding. The EncA protein is highly thermostable, with a *T*_m_ of 76 ± 1 °C, similar to the encapsulin from *Quasibacillus thermotolerans* [28]. The interaction with pUC19 slightly increased the melting temperature by up to 3 °C. A significant difference was found for the value of the enthalpy change at the unfolding transition, ∆*H*_m_, which increased approximately 4-fold in the presence of the DNA (from 45 to 184 kcal/mol). Two phenomena can contribute to raise the ∆*H*_m_ value. It is expected that the number of non-covalent bonds becomes higher due to the formation of new protein–protein and protein–DNA molecular interactions, as seen from AFM results. Additionally, in the presence of DNA, the SRCD spectrum of the fully denatured state is typical of unordered polypeptide chains, which will lead to less favorable hydration of this state. Taken together, during unfolding, the DNA–protein system goes from a state with many non-covalent bonds to fewer when compared to the protein by itself, which can explain the 4-fold increase obtained for ∆*H*_m_.

*M. xanthus* is used as a model organism to study the cooperative mobility and cell-to-cell signaling during multicellular development [10]. Under stress conditions, *M. xanthus* triggers a multicellular development cycle in which the individual rod-shaped cells aggregate to form fruiting bodies, followed by spore development. Kim et al. showed that ∆*encA* (and ∆*encF*) gene deletion in *M. xanthus* impaired the formation of the fruiting body, as the cells became unable to produce DKxanthene and myxoviresin, which are essential for this cell adaptation. The cells were also impaired to agglutinate [32]. The authors correlated this ∆*encA* phenotype with cellular inability to sense iron availability. However, the ∆*encC* and ∆*encD* mutants, with deleted genes encoding putative ferritin-like cargo proteins, maintained the proper transition to fruiting body formation. Following the observations by Kim et al., an additional layer of understanding of this phenotype might be the hitherto uncharacterized ability of EncA to bind and protect DNA against external stresses. Besides being a particularly large iron storage protein that prevents the formation of reactive oxygen species through Fenton chemistry, EncA may also be part of the cellular defense response by DNA protection upon unfavorable conditions, possibly enabling the progress of the mycobacterial life cycle. Thus, EncA may represent a new member of the nucleoid-associated protein family, inducing changes in the DNA topology that allow its protection under stress [29].

Further work is needed to understand if this DNA binding and protection capacity is limited to the *M. xanthus* encapsulin protein and confirm the protection mechanism and physiological relevance of these findings through in vivo studies.

## 4. Materials and Methods

### 4.1. Gene Cloning, Protein Expression and Purification

The *Myxococcus (M.) xanthus* encapsulin protein (EncA) (GenBank ABF87797.1) and its native cargo protein (EncC) (ABF92698.1) were produced in *Escherichia (E.) coli* by recombinant expression. The plasmids harboring the genes coding for both proteins were obtained by chemical synthesis (Invitrogen GeneArt Gene Synthesis, Thermo Fisher Scientific, Waltham, MA, USA). The *encA* gene was subcloned into the pET21-c expression vector (Novagen, MERCK, Darmstadt, Germany) using EcoRI and NdeI enzymes, while the *encC* gene was inserted into pET28-c (Novagen, MERCK, Darmstadt, Germany) using EcoRI, SalI, and T4 DNA ligase through recombinant DNA technology. NZY5α competent cells (NZYTech, Lisbon, Portugal) were transformed with the ligation reactions. Positive clones were isolated from an LB-agar plate containing 100 mg/L ampicillin for pET-21c-EncA transformants or containing 50 mg/L kanamycin for the EncC pET-28c-EncC and grown in liquid LB medium supplemented with the proper antibiotic for plasmid DNA isolation.

The pET-21c-EncA and pET-28c-EncC expression vectors were used to transform *E. coli* BL21(DE3) competent cells (NZYTech, Lisbon, Portugal) for expression of both proteins separately and for co-expression of the EncAC complex. Bacteria harboring the expression vectors were grown in LB medium (25 g/L, NZYTech Lisbon, Portugal) supplemented with the appropriate antibiotics at 37 °C, up to an OD_600 nm_ of about 0.8 and induced with 0.5 mM of IPTG (isopropyl β-D-1-thiogalactopyranoside) overnight at 22 °C, with orbital shaking at 220 rpm. The cells were harvested by centrifugation at 11,000× *g* for 15 min at room temperature (Z 36 HK, HERLME LaborTechnik, Wehingen, Germany) and the pellet was resuspended in 10 mM Tris-HCl buffer pH 7.6 and lysed using an ultrasonic homogenizer (LabsonicM, Sartorius, Goettingen, Germany) in the presence of protease inhibitors (1 mM PMSF and 10 mM Benzamidine) and DNase I (Merck, Darmstadt, Germany). The cellular extract was centrifuged at 11,000× *g* for 20 min at 8 °C to remove cell debris. In the case of EncC, an additional ultracentrifugation step was performed at 186,000× *g* (Beckman Coulter type 45 Ti rotor, Brea, CA, USA) for 1 h at 4 °C. The resulting supernatants were dialyzed overnight against 10 mM Tris-HCl buffer, pH 7.6, and then purified to homogeneity through ionic exchange chromatography and size exclusion steps. All purification steps were performed at 4 °C using an ÄKTA prime plus system (Cytiva, Marlborough, MA, USA).

EncA and EncAC protein extracts were loaded into a cation exchange CM Sepharose Fast Flow column (XK 26/10, Cytiva, Marlborough, MA, USA) pre-equilibrated with 10 mM Tris-HCl buffer, pH 7.6 at a flow rate of 5 mL/min. Both proteins were collected in the flow-through fractions. After dialysis against 10 mM Tris-HCl buffer, pH 7.6, the flow-through fraction was then loaded into a DEAE-Sepharose Fast Flow column (XK 26/40, Cytiva, Marlborough, MA, USA) pre-equilibrated with 10 mM Tris-HCl buffer, pH 7.6 and again eluted as the flow-through at a flow rate of 5 mL/min. The fractions containing EncA (or EncAC) protein were pooled, concentrated using a Vivacell (MWCO 100 kDa, Sartorius, Goettingen, Germany) and loaded into a HiPrep Sephacryl S-500 HR (XK 16/60 cm, Cytiva, Marlborough, MA, USA) previously equilibrated with 200 mM MOPS buffer, pH 7.0 containing 200 mM NaCl. This last purification step yields pure homogenous recombinant EncA or EncAC complex solutions, with typical yield of 100 mg of pure protein per liter of culture.

EncC protein extracts were loaded into a DEAE-Sepharose Fast Flow chromatography column (XK 26/40 cm, Cytiva, Marlborough, MA, USA) pre-equilibrated with 10 mM Tris-HCl buffer, pH 7.6 at a flow rate of 5 mL/min. After a washing step, a linear gradient was applied (0–500 mM NaCl in 10 mM Tris-HCl buffer, pH 7.6) at the same flow to elute adsorbed proteins. Fractions containing the recombinant EncC were pooled, concentrated in a Vivaspin 20 (MWCO 10 kDa, Sartorius, Goettingen, Germany) and applied into a Superdex 200 prep grade size exclusion chromatography column (XK 16/60 cm, Cytiva, Marlborough, MA, USA) previously equilibrated with 200 mM MOPS buffer, pH 7.0, and 200 mM NaCl. This last purification step yields a pure homogenous recombinant EncC solution with a typical yield of 60 mg of pure protein per liter of culture.

Protein fractionation and purity assessment was carried out using 12.5% polyacrylamide SDS-PAGE gels. The LMW II protein marker (NZYTech, Lisbon, Portugal) was used as protein marker.

### 4.2. Protein Quantification and Cargo Loading Determination

The concentrations of EncA and EncC proteins were determined by measuring the absorbance at 280 nm and using the molar absorptivity determined by amino acid sequence analysis (ExPASy ProtParam online tool; Swiss Institute of Bioinformatics, Lausanne, Switzerland) [33]. All measurements were performed on a Thermo Scientific Evolution 210 or 300 UV-Visible spectrophotometers (Thermo Fisher Scientific, Waltham, MA, USA). Standard quartz cells with 1 cm pathlength were used (SUPRASIL, Hellma GmbH, Müllheim, Germany).

Serial dilutions of EncA and EncC samples were performed to determine the relative ratio of EncA and EncC in the EncAC stock solutions using 12.5% polyacrylamide SDS-PAGE gels. The densitometric analysis of the electrophoretic bands was performed using Fiji/ImageJ [34].

### 4.3. Encapsulin Size Determination

Dynamic light scattering (DLS) measurements were made on a HORIBA SZ100 (HORIBA, Kyoto, Japan) equipped with a 10 mW 532 nm laser at a scattering angle of 90°, at 25 °C, for 30 s, in triplicates. Raw data were analyzed using the equipment built-in software assuming a polydisperse sample, a particle refractive index of 1.6 (organic sample), and water settings as dispersion medium (index of 1.333). Samples with 0.5 mg/mL of EncA and EncAC complex were centrifuged at 11,000× *g* for 15 min in 200 mM MOPS buffer, pH 7.0, 200 mM NaCl before each measurement.

### 4.4. Electrophoretic Mobility Shift Assays

Electrophoretic mobility shift assays (EMSAs) were performed using supercoiled pUC19 plasmid in 50 mM MOPS buffer, pH 7.0, 50 mM NaCl (designated as low ionic strength buffer) and in 200 mM MOPS buffer, pH 7.0, 200 mM NaCl (termed as high ionic strength buffer).

The plasmid pUC19 was isolated from *E. coli* DH5α cultures (from cells transformed with a pUC19 stock from the laboratory) using the NZYMidiprep kit and following the manufacturer’s instructions (NZYTech, Lisbon, Portugal). Pure preparations were analyzed by electrophoresis in 1% agarose gels (see Figure S1, Supplementary Materials) and quantified using a NanoDrop 1000 spectrophotometer (ThermoFisher Scientific).

Proteins and pUC19 were dialyzed against the binding buffer for 30 min using a Slide-A-Lyzer mini device (Thermo Fisher Scientific, Waltham, MA, USA) immediately before the binding reaction.

A serial dilution set of EncA and EncAC samples between 1.86 and 0.02 μM and EncC between 336 and 3.88 µM were incubated with 5 nM of pUC19 in a total volume of 20 μL, using both high and low ionic strength buffers for 30 min at room temperature. Next, 1 μL of GreenSafe stain (NZYTech, Lisbon, Portugal) was added to each sample, and 10 μL were loaded into a 1% agarose gel in TAE buffer (40 mM Tris-acetate buffer, pH 8.0, 0.1 mM EDTA), run at 80 V for about 1 h at room temperature. Alternatively, the gels were stained post-running by immersion in a GreenSafe staining solution as per the manufacturer’s protocol. The gels were imaged using a Safe Imager™ transilluminator (Invitrogen, ThermoFischer Scientific, Waltham, MA, USA) and a Gel Logic 100™ Imaging System (Kodak, Rochester, NY, USA). The relative amounts of free and protein−DNA complexes were quantified by measuring the disappearance of the supercoiled conformation using Fiji/ImageJ [34]. The formation of the protein–DNA complexes was plotted against protein concentration and fitted with a Hill equation:

(1) $$f=\frac{f_{\max}\;{\left[\mathrm{Protein}\right]}^{n}}{\left(K_{D\;}+{\left[\mathrm{Protein}\right]}^{n}\right)}$$

where *f* is the fraction of saturation, *f*_max_ corresponds to 100% complex formation, [Protein] is the concentration of protein available to bind, *n* is the Hill coefficient, and *K*_D_ is the macroscopic apparent dissociation constant that corresponds to a measure of the affinity of the protein to the DNA.

The controlled digestion assays were performed using EncA proteins (1.24 µM) pre-incubated with supercoiled pUC19 (5 nM) in both low and high ionic strength buffer. After pre-incubation with DNA, 1.2 µg/µL of DNase I (Merck, Darmstadt, Germany) and 1 mM of MgCl_2_ were added to the samples. The reaction was stopped at different reaction times (0.5, 1, 15, and 30 min) by adding 5 mM of EDTA. The same procedure was performed for free pUC19 samples. The samples were stained with GreenSafe (NZYTech, Lisbon, Portugal) and analyzed by electrophoresis using 1% agarose gels in TAE buffer.

### 4.5. Atomic Force Microscopy Imaging

EncA and EncAC proteins (both free and incubated with pUC19 at a stoichiometry of 1:1 (1.5 nM) at room temperature for 30 min in 50 mM MOPS buffer, pH 7.0 and 50 mM NaCl containing 5 mM NiCl_2_) were used for atomic force microscopy (AFM) imaging. The samples were deposited onto a fresh cleaved grade V1 muscovite mica (Ted Pella, Inc., Redding, CA, USA), incubated for 10 min, washed with deionized water, and dried with a weak stream of nitrogen gas. AFM images were collected in air at room temperature and atmospheric pressure, using an Asylum Research MFP-3D standalone (Oxford Instruments, High Wycombe, UK) operated in alternate contact mode with commercial silicon cantilevers (Olympus AC160TS, f0 = 300 kHz; k = 26 N/m, Olympus Corporation, Tokyo, Japan). Images were processed using the Gwyddion modular program (Czech Metrology Institute, Brno, Czech Republic).

### 4.6. Synchrotron Radiation Circular Dichroism Analysis

Synchrotron radiation circular dichroism (SRCD) spectra were recorded on the AU-CD beam line at the ASTRID2 synchrotron radiation source (ISA, Aarhus University, Aarhus, Denmark). The spectra were obtained in triplicate with 1 nm steps for a wavelength range of 170–280 nm and a dwell time of 2 s per step. The pathlength of the quartz cell (SUPRASIL, Hellma GmbH, Müllheim, Germany) was determined, via an interference technique [35], to be 0.01008 cm. Samples contained EncA at 1.0 mg/mL both with and without pUC19 in a 1:1 molar equivalent stoichiometry, prepared as previously described in 10 mM MOPS buffer, pH 7.0 and 240 mM NaF. Temperature scans were acquired from 4.8 to 83 °C at either 5 or 10 °C increment steps. Molar circular dichroism ∆*ε* was determined from the protein concentration estimated using the absorbance at 205 nm [36]. The secondary structure content was evaluated using DichroWeb analysis tools [26], with CDSSTR as the analysis program and using the SP175 dataset as reference. The melting temperature (*T*_m_) was determined assuming a two-state thermal denaturation model and using a nonlinear least-squares fit procedure using the following equation:

(2) $$\mathrm{SI}\;=\frac{\left(\alpha_{N}+\beta_{N}\times T\right)+\left(\alpha_{D}+\beta_{D}\times T\right)e^{-\frac{\Delta H_{m}\;\left(1-\frac{T}{T_{m}}\right)}{RT}}\;}{\begin{array}{c} 1+e^{-\frac{\Delta H_{m}\;\left(1-\frac{T}{T_{m}}\right)}{RT}}\; \end{array}}$$

∆*H*_m_ is the enthalpy change at the unfolding transition midpoint (kcal/mol), *T*_m_ is the melting temperature, *T* is the temperature in Kelvin, *R* is the universal gas constant (1.987 cal/K/mol), and $\alpha_{N}$, $\beta_{N}$, $\alpha_{D}$, and $\beta_{D}$ taking into account ∆*ε* as well are the baseline slope and intercept pre-transition and post-transition baseline, respectively.

## Figures and Tables

**Table 1:** Secondary structure composition of the EncA complex by deconvolution of the SRCD spectra at 25 °C.

		Secondary Structure Content (%)		
		*α*-Helix	*β*-Sheet	Others
EncA	DichroWeb	26.4 ± 0.5	26.0 ± 0.2	46.0 ± 0.1
	2Struc (4PT2)	25.1	15.0	59.9
	2Struc (7S20)	27.2	15.3	57.5

## Data Availability

Not applicable.

## Supplementary Materials

The supporting information can be downloaded at: https://www.mdpi.com/article/10.3390/ijms23147829/s1.
