# Supplementary material for: Condensation and Protection of DNA by the Myxococcus xanthus Encapsulin: A Novel Function
Source: Int J Mol Sci. 2022 Jul 15;23(14):7829. doi: 10.3390/ijms23147829 (PMC9321382; doi:10.3390/ijms23147829)
Supplement: Supplementary file 1 [file ijms-23-07829-s001.zip › ijms-1730841-supplementary.pdf]

International Journal of
Molecular Sciences

MDPI

Supplementary Materials

Condensation and Protection of DNA by the *Myxococcus xanthus* Encapsulin: A Novel Function

Ana V. Almeida[1,2], Ana J. Carvalho[1,2], Tomás Calmeiro[3], Nykola C. Jones[4], Søren V. Hoffmann[4], Elvira Fortunato[3], Alice S. Pereira[1,2,*] and Pedro Tavares[1,2,*]

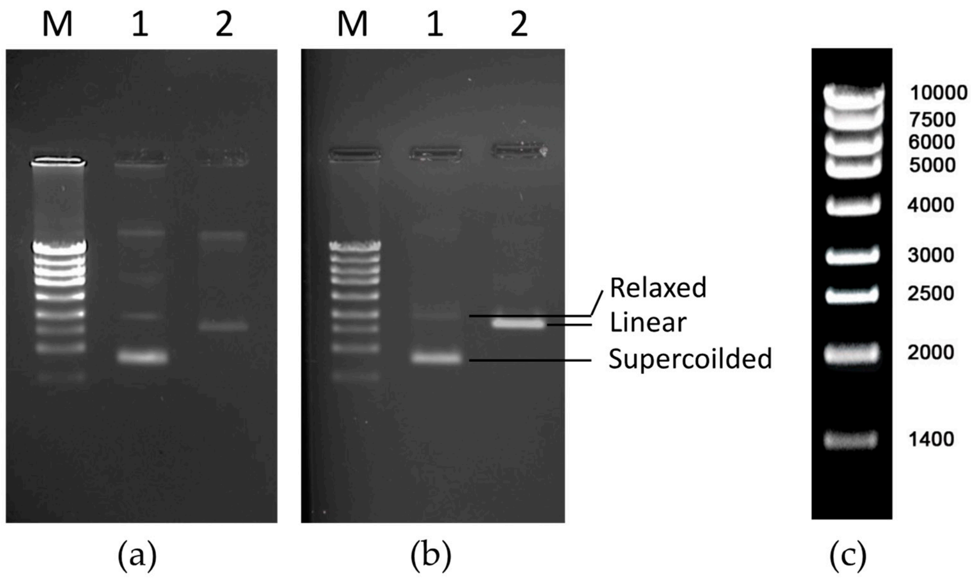

Figure S1. pUC19 in different conformations 50 mM MOPS pH 7.0 buffer containing 230 mM NaCl and comparison of gel staining procedures. 1 – As-isolated pUC19; 2 – Linear pUC19 after digestion with 10 U of EcoRI (NZYTech) for 1 h, at 37 °C; M – NZYDNA Ladder II. In (a) the GreenSafe dye was added to the samples before loading into the gel, whereas in (b), the gel (with the exact same samples) was stained post-electrophoresis in a GreenSafe bath. Duplicated samples were applied in two halves on a single gel, run at 80 V for 1 h, which was then cut into halves for band visualization. (c) Band separation of the NZYDNA Ladder II in 1 % agarose gel from the manufacturer's protocol (catalogue number MB04301, https://www.nzytech.com/products-services/molecular-biology/ladders-markers/dna-ladders/mb043/). While not exactly the same, the results show that the staining procedure does not significantly affect the visualization of the bands. The supercoiled, linear and relaxed forms of the plasmid are identified on the right. Above the linear form, bands with lower intensity are observed corresponding to the open circle forms with different numbers of nicks.

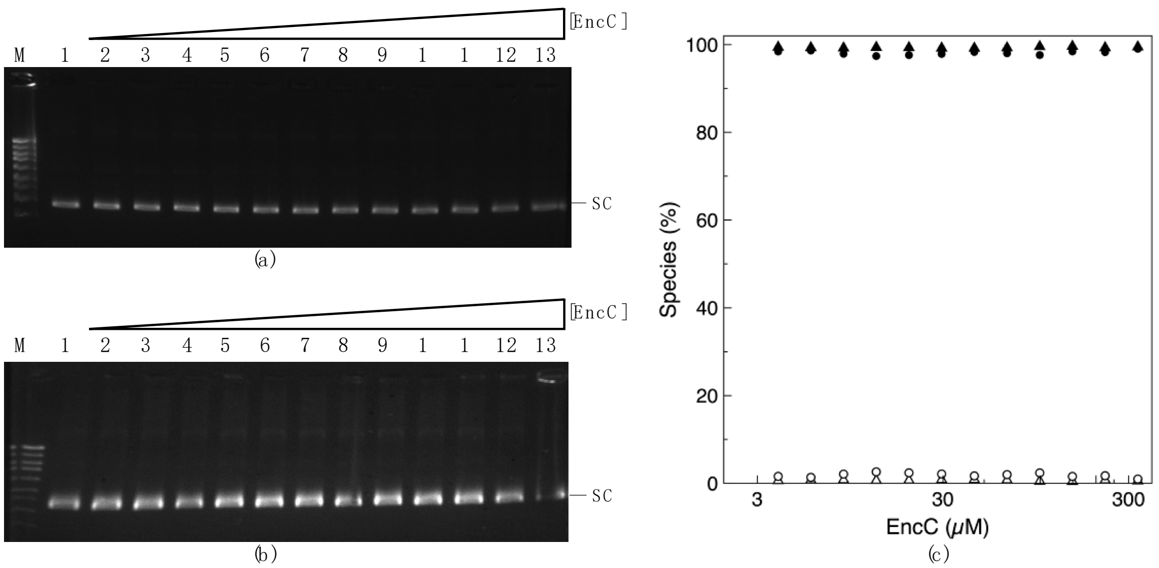

Figure S2. Binding of the cargo protein, EncC, to supercoiled pUC19 (5 nM) at high and low ionic strength conditions. (a) EMSA of EncC in 50 mM MOPS buffer, pH 7.0, 50 mM NaCl and (b) 200 mM MOPS buffer pH 7.0, 200 mM NaCl. M – NZYLadder II; 1 to 13—Binding of EncC to the plasmid DNA with increasing protein concentrations: 0, 3.88, 5.8, 8.7, 13.1, 19.7, 29.5, 44.2, 66.4, 99.6, 149, 224 and 336 μM. The free form of the supercoiled plasmid pUC19 band (SC) is labelled. (c) Plots from the densitometric analysis of the three sets of experiments in either 50 mM MOPS buffer pH 7.0, 50 mM NaCl (circles) and 200 mM MOPS buffer, pH 7.0, 200 mM NaCl (triangles). The free DNA is plotted as full markers, and the protein-DNA complex as empty circles or triangles.

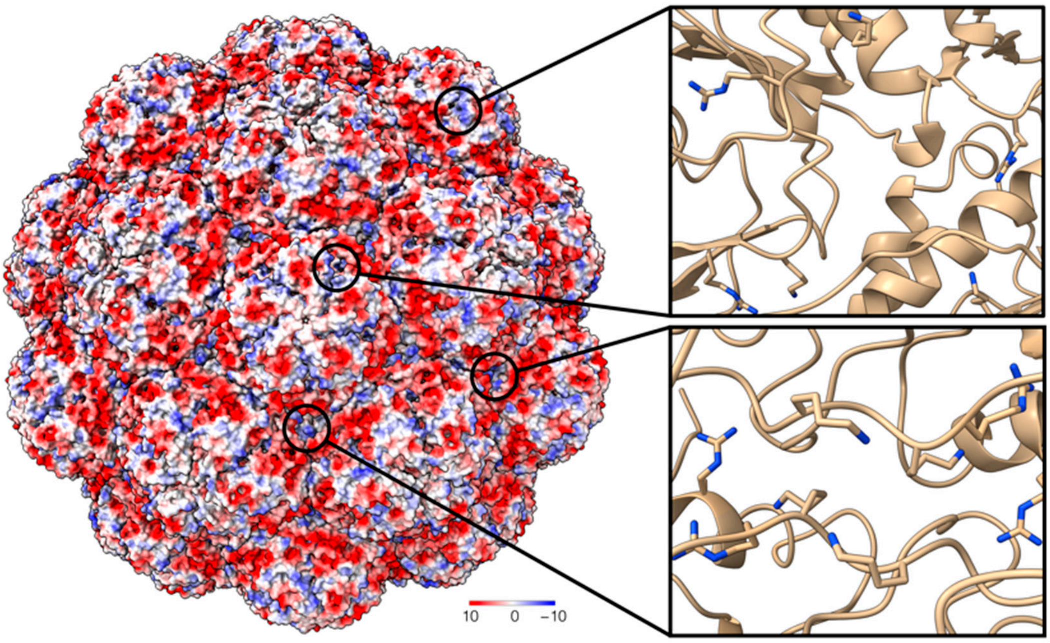

Figure S3. Electrostatic surface potential representation of the *M. xanthus* encapsulin protein shell. The positively charged pockets (arginine and lysine residues) are magnified and displayed as sticks on the right side. The electrostatic surface potentials are colored with a range from red (negative) to white (neutral) to blue (positive) corresponding to a +10 kTe^{-1} to 0 to −10 kTe^{-1}.
